# Effects of Exercise Training on Experimental Chronic Kidney Disease: A Systematic Review and Meta-Analysis

**DOI:** 10.3390/ijms27177830

**Published:** 2026-09-01

**Authors:** Muheebur Rehman, Iqbal Ali Shah, Kun-Ling Tsai, Shin-Da Lee, Bor-Tsang Wu

**Affiliations:** 1 PhD Program in Healthcare Science, College of Healthcare Science, China Medical University, Taichung 406040, Taiwan; u114313102@cmu.edu.tw (M.R.); u112313102@cmu.edu.tw (I.A.S.); 2 Department of Physical Therapy, College of Medicine, National Cheng Kung University, Tainan 701401, Taiwan; kunlingtsai@mail.ncku.edu.tw; 3 Department of Physical Therapy, China Medical University, Taichung 406040, Taiwan; 4 Department of Senior Citizen Service Management, National Taichung University of Science and Technology, Taichung 404336, Taiwan

**Keywords:** apoptosis, chronic kidney disease, exercise training, oxidative stress, inflammation

## Abstract

Chronic kidney disease (CKD) promotes progressive renal dysfunction through a complex interplay of oxidative stress, chronic inflammation, apoptosis, and metabolic disturbances. This systematic review and meta-analysis aimed to evaluate the effects of exercise training on systemic adaptations as well as tissue-specific renal and muscular mechanisms in CKD rat models. In accordance with the Preferred Reporting Items for Systematic Reviews and Meta-Analyses (PRISMA) 2020 guidelines, PubMed, Embase, and Web of Science were searched from 2000 to 2026. Of 1287 records, 11 studies were included in the systematic review, of which 8 studies were evaluated in the meta-analysis. Methodological quality was assessed using the Collaborative Approach to Meta-Analysis and Review of Animal Data from Experimental Studies (CAMARADES) checklist, with scores ranging from 5 to 7 out of 10. Exercise interventions included resistance training, treadmill running, aerobic exercise, and wheel climbing. Exercise training enhanced endothelial function and improved metabolic homeostasis while attenuating reactive oxygen species (ROS) generation and lipid peroxidation through augmentation of endogenous antioxidant defenses. Restoration of redox balance suppressed nuclear factor kappa B (NF-κB) and NOD-, LRR-, and pyrin domain-containing protein 3 (NLRP3) inflammasome signaling pathways, resulting in reductions in pro-inflammatory cytokines, including tumor necrosis factor-alpha (TNF-α), interleukin-1 beta (IL-1β), and interleukin-6 (IL-6). These effects subsequently mitigated mitochondrial dysfunction, caspase activation, and apoptosis-related signaling, thereby preserving renal cellular viability. These adaptations translated into substantial histopathological protection, characterized by reduced inflammatory cell infiltration, glomerular sclerosis, tubular degeneration, and extracellular matrix deposition. Preservation of renal microarchitecture contributed to improved filtration capacity and overall renal function. Furthermore, exercise promoted mitochondrial biogenesis and anabolic signaling in skeletal muscle via activation of peroxisome proliferator-activated receptor gamma coactivator 1-alpha (PGC-1α)/transcription factor A, mitochondrial (TFAM) pathways, enhancing protein synthesis and attenuating CKD-associated muscle wasting, thereby contributing to tissue-level protection and systemic physiological improvement.

## 1. Introduction

Chronic kidney disease (CKD) is a progressive disorder characterized by persistent structural and functional abnormalities of the kidneys, resulting in reduced glomerular filtration and impaired fluid, electrolyte, metabolic, and endocrine homeostasis [1]. As renal function declines, waste metabolites such as urea and creatinine accumulate in the circulation, contributing to widespread systemic complications [2,3]. CKD affects approximately 10% of the global population and has emerged as a leading cause of mortality worldwide. In Asia, CKD prevalence more than doubled from 202.4 million cases in 1990 to 431.2 million in 2019, with over 760,000 new cases reported in 2019 [4]. Although CKD is traditionally defined by renal dysfunction, it is now widely documented as a multisystem disorder affecting nearly every organ system [5].

In addition to reduced glomerular filtration rate (GFR), CKD frequently causes proteinuria, anemia, dyslipidemia, mineral and bone disorders, hypertension, metabolic acidosis, skeletal muscle wasting, endothelial dysfunction, and impaired physical capacity [6,7]. These complications substantially increase the risks of disability and mortality, highlighting the need for therapeutic strategies targeting both renal injury and systemic dysfunction [8]. CKD pathophysiology is multifactorial and involves interconnected processes that promote progressive nephron loss. Initial injury caused by diabetes, hypertension, toxins, autoimmune disease, or nephron reduction triggers glomerular hypertension, tubular dysfunction, inflammation, apoptosis, and fibrosis [9,10]. Over time, these pathological responses promote glomerulosclerosis, tubular atrophy, tubulointerstitial fibrosis, and irreversible destruction of renal architecture, ultimately leading to progressive loss of kidney function [11].

Among the major drivers of CKD progression, oxidative stress is considered particularly important. Oxidative stress occurs when the generation of reactive oxygen species (ROS) exceeds the capacity of endogenous antioxidant defense systems [12]. In CKD, mitochondrial dysfunction, nicotinamide adenine dinucleotide phosphate (NADPH) oxidase activation, xanthine oxidase activity, and retention of uremic toxins markedly increase ROS production [13]. Excessive ROS damages lipids, proteins, and DNA, impairs nitric oxide (NO) bioavailability, and exacerbates endothelial and microvascular dysfunction. Simultaneously, antioxidant defense systems such as superoxide dismutase (SOD), catalase, glutathione peroxidase (GPx), and total thiol reserves are frequently diminished, further aggravating cellular injury [12,14]. Chronic low-grade inflammation closely interacts with oxidative stress pathways [15]. Elevated circulating and tissue concentrations of tumor necrosis factor-alpha (TNF-α), interleukin-6 (IL-6), interleukin-1 beta (IL-1β), transforming growth factor-beta (TGF-β), interferon-gamma (IFN-γ), and other inflammatory mediators contribute to leukocyte recruitment, fibroblast activation, collagen deposition, and vascular dysfunction [16,17]. Persistent inflammatory signaling through pathways such as nuclear factor kappa B (NF-κB), mammalian target of rapamycin (mTOR), toll-like receptors, and inflammasome activation further accelerates renal fibrosis and systemic catabolism [17,18]. Together, oxidative stress and inflammation create a self-perpetuating cycle that drives CKD progression.

Besides renal injury, CKD profoundly affects skeletal muscle structure and function [19]. Muscle wasting, weakness, reduced mitochondrial biogenesis, impaired energy metabolism, decreased exercise tolerance, and sarcopenia are common findings in both patients and experimental CKD models [20]. These abnormalities are mediated by inflammation, insulin resistance, metabolic acidosis, inactivity, and activation of proteolytic pathways such as MuRF-1, Atrogin-1, and myostatin signaling. Loss of muscle mass and physical function significantly worsens quality of life and is independently associated with mortality in CKD populations [20,21]. Exercise training is a promising non-pharmacological intervention with potential renoprotective, anti-inflammatory, antioxidant, cardiovascular, and anabolic effects [22]. Regular physical activity improves endothelial function, blood pressure regulation, insulin sensitivity, mitochondrial efficiency, muscular strength, and aerobic capacity. In clinical CKD populations, structured exercise programs have been associated with improved functional status, reduced inflammation, and better health-related quality of life [23,24]. Experimental studies using treadmill running, swimming, voluntary wheel running, and resistance ladder climbing have further demonstrated reductions in serum urea, creatinine, blood urea nitrogen (BUN), proteinuria, oxidative damage, inflammatory cytokines, fibrosis, and apoptosis, alongside improvements in GFR, muscle function, body composition, and renal structural preservation [25,26].

Despite these encouraging findings, the literature remains heterogeneous. Differences in animal species, CKD induction methods, exercise modality, training intensity, session duration, intervention length, and outcome measures make it difficult to establish definitive conclusions regarding the efficacy of exercise in experimental CKD [27]. Furthermore, although numerous experimental studies demonstrate the potential benefits of exercise, comprehensive synthesis evaluating the collective effects of exercise training on renal parameters, oxidative stress biomarkers, inflammatory mediators, and tissue-specific outcomes remains limited [28]. Therefore, the current systematic review aims to critically evaluate and synthesize the effects of exercise training on renal functional parameters, oxidative stress markers, inflammatory biomarkers, and tissue-specific outcomes in experimental CKD animal models to better elucidate underlying mechanisms and inform future therapeutic strategies.

## 2. Methods

### 2.1. Protocol and Registration

This systematic review and meta-analysis was conducted in accordance with Preferred Reporting Items for Systematic Reviews and Meta-Analyses (PRISMA) guidelines. The review was registered in the International Platform of Registered Systematic Review and Meta-Analysis Protocols (INPLASY) (Reg No. INPLASY202640050).

### 2.2. Information Sources

A systematic literature search was conducted from three databases: PubMed, Web of Science, and EMBASE to identify eligible studies published between 2000 and 2026. Additional studies were identified through manual screening of reference lists from eligible articles.

### 2.3. Eligibility Criteria

Eligibility criteria were defined according to the Population, Intervention, Comparator, Outcome, and Study design (PICOS) framework.

#### 2.3.1. Animal Model Type

Studies using rat or mouse models of CKD induced by surgical (e.g., 5/6 nephrectomy), chemical (e.g., doxorubicin), or genetic methods were included. Studies that used healthy animals, non-CKD models, or in vitro experiments were excluded. Information regarding animal species, strain, sex, age, body weight, and CKD induction method was extracted where available.

#### 2.3.2. Types of Intervention

Interventions comprised structured exercise protocols such as aerobic, interval, and resistance training implemented via treadmill running, ladder climbing, swimming, or motorized wheels, with a minimum duration of three weeks. Moreover, studies evaluating exercise combined with pharmacological agents, dietary supplementation, or other non-exercise interventions were excluded.

#### 2.3.3. Comparator

Comparator groups consisted of sedentary animals (rats/mice) with CKD that did not receive any exercise intervention.

#### 2.3.4. Study Outcomes

Primary outcomes included renal function, oxidative stress, apoptosis, inflammatory and molecular signaling pathways, and kidney histopathology. Renal outcomes included blood urea nitrogen (BUN), serum creatinine, proteinuria, creatinine clearance, estimated GFR, and plasma electrolytes. Oxidative stress and antioxidant markers included superoxide dismutase (SOD, SOD2), catalase (CAT), glutathione peroxidase (GPx), malondialdehyde (MDA), nitric oxide (NO), protein carbonyls, NADPH oxidase, and xanthine oxidase activity. Apoptotic outcomes included Bax/Bcl-2 ratio, caspase activity, μ-calpain, DNA fragmentation, and TUNEL-positive cells. Inflammatory and molecular markers included interleukins, transforming growth factor-beta (TGF-β), Fas (CD95), heme oxygenase-1 (HO-1), inducible nitric oxide synthase (iNOS), and NLRP3 inflammasome components. Histopathological outcomes included glomerular injury, tubulointerstitial fibrosis, and preservation of renal cortical architecture.

Secondary outcomes included systemic physiological and muscular parameters such as body weight, kidney weight, kidney-to-body weight ratio, systolic blood pressure, muscle mass, grip strength, maximal load capacity, muscle fiber cross-sectional area, and myogenic markers.

#### 2.3.5. Study Design

Original controlled animal studies written in English and published between 2000 and 2026 were included. Studies with intervention durations of <3 weeks, non-exercise interventions, or combining exercise with pharmacological or dietary treatments were excluded. Case reports, case series, cross-sectional studies, editorials, and review articles were also excluded.

### 2.4. Search Strategy

Search terms related to chronic kidney disease, exercise training, and animal models were combined using Boolean operators as follows:

(“chronic kidney disease” OR CKD OR “renal insufficiency, chronic” OR “chronic renal failure” OR CRF OR uremia OR uremic OR “kidney failure” OR “renal dysfunction”) AND (“exercise” OR “exercise training” OR “exercise therapy” OR “physical activity” OR “physical fitness” OR “aerobic exercise” OR “resistance training” OR “endurance training” OR “strength training” OR “treadmill training” OR “voluntary exercise” OR “forced exercise” OR “exercise intervention” OR “physical training” OR “motor activity”) AND (“disease models, animal” OR “animal model” OR animals OR rat OR rats OR mouse OR mice OR murine OR rodent OR rodents).

### 2.5. Study Selection

After the removal of duplicate records, two reviewers independently screened titles and abstracts for eligibility. Full-text articles of potentially relevant studies were subsequently screened according to the predefined inclusion criteria. Disagreements were resolved through discussion with a third reviewer.

### 2.6. Data Extraction

Two reviewers independently extracted data, which included study characteristics, animal model, intervention characteristics, comparator groups, and outcome measures. Data were extracted from the methods, results, tables, and figures of the included studies.

### 2.7. Data Management

All the extracted data were managed in Microsoft Excel spreadsheets. Tables, figures, and manuscript drafts were stored in separate files and regularly updated throughout the review process to ensure accurate documentation and version control. The extracted data are available in Supplementary File S1.

### 2.8. Data Items

Data items obtained from each study included: last name of first author, publication year, animal species, sex, age, body weight, CKD induction method, exercise modality, exercise intensity, frequency, duration, comparator groups, sample size, renal function outcomes, oxidative stress markers, inflammatory and molecular markers, histopathological findings, skeletal muscle outcomes, and data required for quantitative synthesis. No assumptions regarding missing outcome data were made.

### 2.9. Risk of Bias Assessment

Risk of bias was independently assessed by two reviewers using the Collaborative Approach to Meta-Analysis and Review of Animal Data from Experimental Studies (CAMARADES) 10-item checklist for preclinical animal studies.

The CAMARADES assessment of the 11 included studies demonstrated methodological quality scores ranging from 5 to 7/10. All studies were peer-reviewed and complied with regulatory requirements, and most reported temperature control and randomization of experimental groups. However, allocation concealment was consistently absent, and blinded outcome assessment was limited. Reporting of anesthetic use and appropriateness of the animal model was inconsistent, and none of the studies reported sample size calculations, which might indicate potential risk of bias. The detailed CAMARADES assessment is presented in Table 1.

### 2.10. Data Collection Process

**Figure 1:**
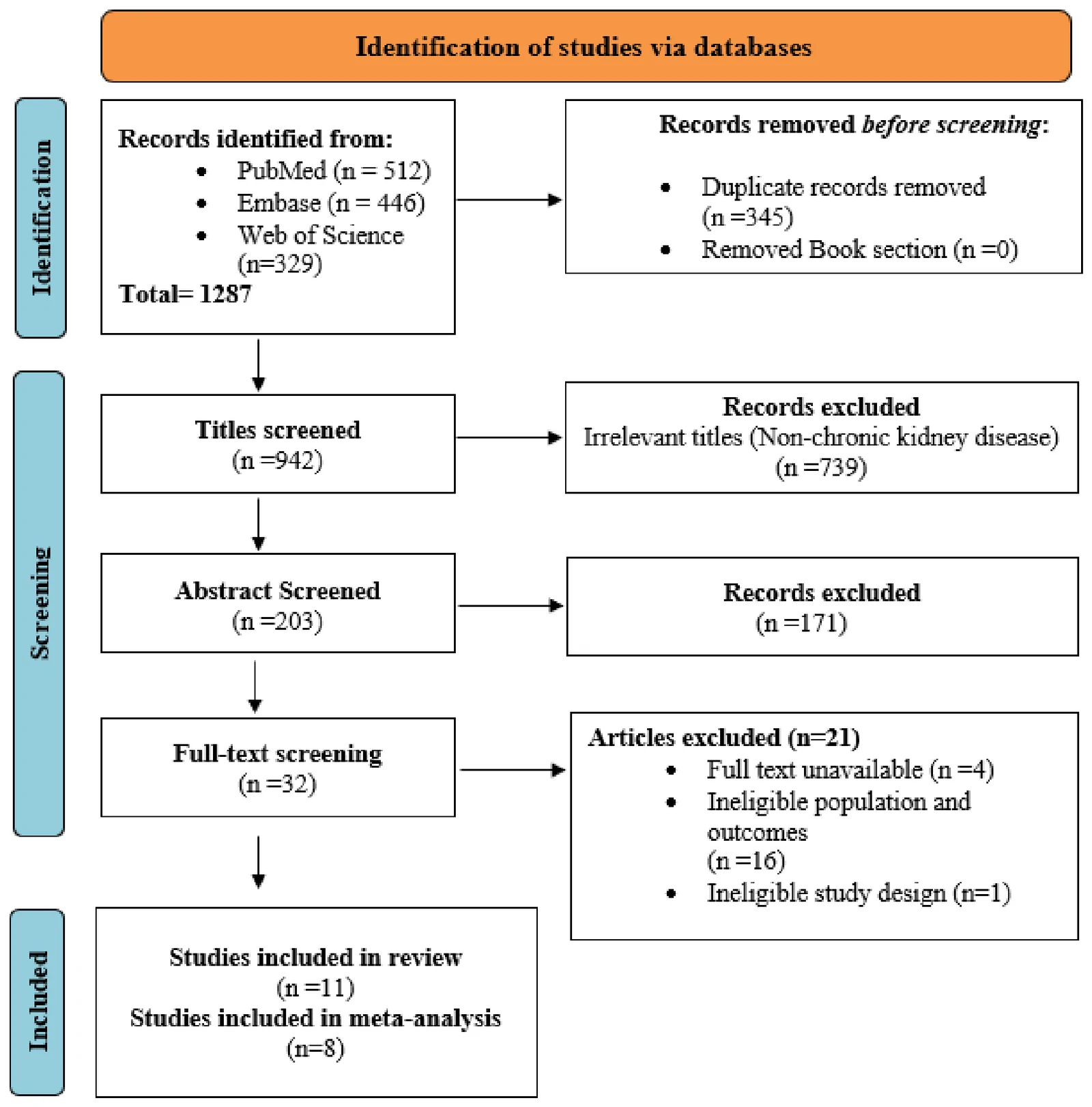
PRISMA flow diagram of the study selection process.

The Data Collection Process is presented in the PRISMA flow chart (Figure 1). The characteristics of the included studies were: the name of the first author, year of publication, type of animal model, exercise type, and type of CKD induced. The effect of exercise on systemic outcomes and tissue-specific outcomes was indicated in the text, tables, and mechanistic figure using the signs of increase (↑), decrease (↓), and no change (↔).

### 2.11. Data Synthesis

Extracted data were organized in Microsoft Excel before synthesis. Continuous outcomes reported using different measurement scales were standardized by calculating standardized mean differences (SMDs). Study findings and characteristics were presented in tabulated form. Forest plots were generated to display pooled effect estimates for the meta-analysis.

### 2.12. Effect Measures

For quantitative analysis, continuous outcomes were summarized as standardized mean differences (SMDs) with 95% confidence intervals (CIs).

### 2.13. Statistical Analysis

Statistical analysis was conducted using Review Manager (RevMan version 5.4.1). Meta-analyses were performed separately for renal parameters and oxidative stress markers. Pooled effect sizes were calculated as standardized mean differences (SMDs) with 95% confidence intervals (CIs). A random-effects model was applied because of variability in disease stages, exercise modalities, intervention characteristics, and experimental settings across studies. Heterogeneity was assessed using the I^2^ statistic, with values of 0–40% considered potentially unimportant, 30–60% moderate, 50–90% substantial, and 75–100% considerable heterogeneity [27]. Statistical significance was considered at *p* < 0.05.

Where sufficient data were available, subgroup analysis was performed according to exercise duration to explore whether the effects differed between shorter- and longer-duration exercise interventions. Specifically, studies [29,33] were categorized into 30 min and 60 min exercise-duration subgroups. The subgroup analysis was conducted to explore potential differences in treatment effects according to exercise duration; however, these findings were interpreted cautiously because of the limited number of studies contributing to each subgroup.

In addition, fewer than ten studies contributed to each pooled outcome; therefore, publication bias was not assessed using funnel plots or statistical tests. The certainty of the overall body of evidence was not formally assessed using GRADE or another certainty assessment framework because this review synthesized evidence from preclinical animal studies, for which such assessments were not undertaken.

## 3. Results

### 3.1. Search Results

The initial database search identified 1287 records from PubMed (n = 512), Web of Science (n = 329), and EMBASE (n = 446) (Figure 1). After removal of duplicate records (n = 345), 942 studies remained for title and abstract screening. During screening, 739 records were excluded because they were irrelevant or unrelated to CKD. Consequently, 203 articles were assessed for eligibility, of which 171 were excluded based on the predefined eligibility criteria. The remaining 32 full-text articles were reviewed in detail. Of these, 21 studies were excluded because of unavailable full text (n = 4), ineligible population or outcomes (n = 16), and ineligible study design (n = 1). Ultimately, 11 studies were included in the systematic review [29,30,31,32,33,34,35,36,37,38,39], of which eight were eligible for meta-analysis [29,30,31,32,33,34,35,38].

### 3.2. Biological Specimens Used for Biomarker Assessment

#### 3.2.1. Serum and Plasma Biomarkers

Serum and plasma constituted the principal biological specimens for assessment of circulating renal, metabolic, cardiovascular, oxidative, inflammatory, and endocrine biomarkers. Renal function was evaluated using BUN, urea, creatinine, and urea nitrogen, with these parameters reported in serum or plasma [29,30,32,33,34,35,36,37,38]. Calcium, phosphorus, phosphate, parathyroid hormone (PTH), albumin, cholesterol, triglycerides, glucose, lactate, sodium (Na^+^), potassium (K^+^), and NO were also assessed in blood-derived specimens as systemic physiological or metabolic measures [29,32,33,34,36]. In addition, MMP-2 and MMP-9 were measured in serum/plasma as circulating molecular markers [33]. Oxidative stress in circulating specimens was evaluated using SOD, MDA, and thiobarbituric acid reactive substances (TBARS) [29,33], while inflammatory assessment included circulating IL-6 [33]. In the muscular study, creatine kinase (CK) was specifically measured in serum as a marker associated with muscle injury or muscular adaptation [39]. Thus, serum/plasma specimens encompassed a broad spectrum of renal function, metabolic homeostasis, oxidative status, inflammatory activity, and muscle-related biochemical markers [29,30,31,32,33,34,35,36,37,38,39].

#### 3.2.2. Whole-Blood Biomarkers

Whole blood was used specifically for hematological assessment. The evaluated parameters comprised red blood cell (RBC) count, platelet count, and white blood cell (WBC) count [29]. These measurements represent cellular blood indices rather than soluble biochemical biomarkers and therefore provide complementary information regarding systemic hematological status.

#### 3.2.3. Urinary Biomarkers

Urine was primarily used to characterize renal excretory function, protein loss, and urinary oxidative products. Proteinuria/urinary protein was assessed in 24 h or urine specimens [33,34,37], while urinary BUN was evaluated in the swimming-exercise study [33]. Urinary MDA was also measured in 24 h urine collections [34]. Furthermore, creatinine clearance was derived from serum/plasma and urinary measurements and was used as an indicator of renal filtration [37]. Therefore, urinary specimens provided direct information regarding protein leakage, urinary nitrogen excretion, oxidative products, and filtration-related renal function [33,34,37].

#### 3.2.4. Kidney Tissue and Renal Homogenates

Kidney tissue represented the major specimen for evaluating organ-specific oxidative stress, antioxidant defense, inflammatory signaling, fibrotic pathways, and molecular responses. Renal tissue or kidney homogenates were used to assess superoxide production, SOD, catalase (CAT), GPx, TBARS, MDA, protein carbonyls, and total sulfhydryl/thiol content [30]. Renal tissue was additionally analyzed for MDA, SOD activity, and hydrogen sulfide (H_2_S) [38]. In the renal cortex, NADPH oxidase activity and xanthine oxidase (XO) activity were assessed as enzymatic sources of oxidative stress [34]. Corresponding molecular expression analyses included Nox2, Nox4, and XO in renal cortical tissue [34].

#### 3.2.5. Renal Inflammatory and Molecular Signaling Biomarkers

Renal tissue and renal cortical specimens were also used to characterize local inflammatory, profibrotic, growth-factor, and intracellular signaling pathways. Tissue-based inflammatory biomarkers included IL-10, TNF-α, TGF-β, IL-4, and IL-6 [32,33,37,39]. Molecular signaling markers included mTOR, ribosomal protein S6 (rpS6), PTEN, HO-1, iNOS, PDGFR, phosphorylated PDGFR (p-PDGFR), α-smooth muscle actin (α-SMA), and CD34 [32,33,37]. In particular, PDGFR, p-PDGFR, and α-SMA were assessed in the renal cortex, whereas CD34 was assessed in kidney sections/glomeruli [33]. These tissue-derived biomarkers therefore provide evidence of local inflammatory activation, profibrotic signaling, growth-factor activity, vascular/molecular responses, and intracellular pathway modulation [32,33,37,39].

#### 3.2.6. Renal Mitochondrial and Apoptotic Biomarkers

Subcellular renal specimens were used to investigate apoptosis and mitochondrial mechanisms of kidney injury. A renal mitochondrial fraction was analyzed for intramitochondrial cytochrome c, whereas renal cortex tissue lysates were used to evaluate Bcl-2, caspase-12, μ-calpain, CHOP, GRP78, and Fas/CD95 [29]. Cleaved caspase-3 was assessed in both renal cortex tissue lysates and kidney tissue sections [29,35], while cleaved caspase-8 and cleaved caspase-9 were evaluated in kidney tissue sections [29]. In addition, TUNEL-positive apoptotic cells and DNA fragmentation were assessed in kidney tissue sections [29]. Collectively, these biomarkers were derived from renal cortical tissue, renal mitochondrial fractions, and histological kidney sections, allowing assessment of both intracellular apoptotic signaling and tissue-level cell death [29].

#### 3.2.7. Skeletal Muscle Tissue Biomarkers

Skeletal muscle tissue was used to investigate the muscular consequences of CKD and exercise-related molecular adaptations. Gastrocnemius muscle tissue was analyzed for SOD2 and MDA as oxidative stress markers; IL-6, TNF-α, NLRP3, ASC, caspase-1, IL-1β, and IL-18 as inflammatory and inflammasome-related markers; and Bax and cleaved caspase-3 as apoptotic markers [35]. The same muscle tissue was further assessed for myosin heavy chain (MyHC), MyoD, myogenin, Pax-7, atrogin-1, MuRF-1, myostatin, mitochondrial DNA (mtDNA), ATP, PGC-1α, TFAM, and CoxIV, representing muscle differentiation, regeneration, proteolysis, atrophy, mitochondrial content, energy metabolism, and mitochondrial biogenesis [35].

In EDL muscle tissue, ubiquitin protein expression, Atrogin-1 gene expression, IGF-1 gene expression, p70 signaling, Pax-7 gene expression, MyoD gene expression, and Myogenin gene expression were assessed [36]. Functional and structural muscle outcomes in this tissue included maximal isometric torque, half-relaxation time, muscle fiber cross-sectional area, and protein turnover/regeneration-related measures [36]. In addition, muscle mass was evaluated in the EDL, tibialis anterior, plantaris, and soleus muscles, together with maximal weight-carrying capacity [37]. In another resistance-training study, muscle outcomes included muscle strength and muscle mass, with mass determined from the soleus, gastrocnemius, and quadriceps [39].

#### 3.2.8. Renal Histopathology and Structural Tissue Outcomes

Kidney tissue sections were used for direct assessment of renal structural injury and histopathological alterations. The evaluated structural outcomes included TUNEL-positive apoptotic cells, DNA fragmentation, collagen deposition, glomerular volume, edema, glomerular injury, tubulointerstitial fibrosis, glomerulosclerosis, capsular adhesions, interstitial fibrosis, inflammatory infiltration, tubular atrophy, tubular cell loss, and tubular disintegration [29,33,35,37,38]. These outcomes were derived from kidney tissue sections or renal tissue specimens, providing direct morphological evidence of renal injury, fibrosis, cellular loss, and structural remodeling.

### 3.3. Effect of Exercise Training on Systemic Outcomes in Experimental Chronic Kidney Disease Model

#### 3.3.1. Renal Parameters (Serum/Plasma/Urine)

Across the included studies, CKD consistently induced marked elevations in BUN, creatinine, urea, and proteinuria, reflecting progressive renal dysfunction. Exercise training demonstrated predominantly renoprotective, although heterogeneous, effects across experimental models. Most studies reported significant reductions in BUN and proteinuria following aerobic or resistance exercise interventions [29,32,33,36,38] suggesting improved renal excretory function and attenuation of glomerular permeability impairment. In contrast, findings for serum urea and creatinine were less consistent. Several studies demonstrated significant reductions following exercise interventions [30,32,35], whereas others reported minimal or no changes [31,34]. This variability may reflect differences in CKD severity, residual nephron mass, and the extent of irreversible structural injury across experimental models (Table 2).

#### 3.3.2. Kidney Functional Parameters

Limited studies assessed functional indices; however, available evidence suggests beneficial effects of exercise. Improvements in GFR were reported following resistance training [32], while reductions in kidney weight-to-body weight ratio were observed in aerobic and swimming models [29,33], indicating attenuation of renal hypertrophy (Table 2).

#### 3.3.3. Physiological and Physicochemical Outcomes

Exercise produced consistent improvements in physiological parameters. Blood pressure, lipid profile, lactate levels, and electrolyte balance were favorably modulated [32,33,37,38]. Body weight, which was reduced in CKD animals, improved following exercise [32,33,37], while hematological abnormalities, including RBC, WBC, and platelet disturbances, were partially normalized [29] (Table 2).

#### 3.3.4. Systemic Oxidative Stress and Antioxidant Markers

CKD induced marked oxidative stress, characterized by reduced antioxidant enzyme activity, including SOD, alongside increased oxidative stress markers such as MDA and TBARS. Exercise interventions consistently improved oxidative balance by increasing SOD levels and reducing MDA and TBARS concentrations [29,32,34]. In addition, resistance training enhanced NO availability, further supporting vascular and antioxidant function [32] (Table 2).

#### 3.3.5. Systemic Inflammatory and Molecular Signaling Markers

Limited evidence indicated that CKD was associated with elevated circulating pro-inflammatory cytokines, including IL-6, TNF-α, IFN-γ, and IL-2. Exercise training reduced these inflammatory markers, supporting its anti-inflammatory effects in experimental CKD models [32,33] (Table 2).

### 3.4. Effect of Exercise on Tissue-Specific Outcomes in Experimental Chronic Kidney Disease Model

#### 3.4.1. Tissue-Specific Oxidative Stress and Antioxidant Markers

Across the included studies, CKD was generally associated with a marked disturbance in tissue redox homeostasis, particularly in the kidney and skeletal muscle. In renal tissue, CKD increased superoxide generation, TBARS, protein carbonyls, NADPH oxidase activity, xanthine oxidase (XO) activity, and the expression of Nox2, Nox4, and XO, while antioxidant defenses such as total thiols and SOD were reduced or otherwise dysregulated [30,31,34,38]. These findings indicate that CKD promotes excessive ROS generation and oxidative damage within the kidney. Importantly, exercise training consistently attenuated these abnormalities, with reductions in superoxide, TBARS, protein carbonyls, NADPH oxidase, and XO activity, together with improvements in SOD, GPx, total thiols, and related antioxidant defenses [30,31,34,38]. In skeletal muscle, CKD was similarly characterized by reduced SOD2 and increased MDA, indicating oxidative stress in muscle tissue; exercise reversed these changes by increasing SOD2 and reducing MDA [35]. Thus, the tissue-level evidence suggests that exercise exerts a predominantly antioxidant and redox-restorative effect, although the specific antioxidant response varied between studies and biomarkers [30,31,34,35,38] (Table 3).

#### 3.4.2. Tissue-Specific Inflammatory and Molecular Signaling Markers

Inflammatory and molecular signaling abnormalities were primarily identified in kidney and skeletal muscle tissues. In the kidney, CKD was associated with increased TNF-α, TGF-β, mTOR, and rpS6, together with reduced IL-10 and PTEN, suggesting a pro-inflammatory and dysregulated growth/signaling environment [37]. Similarly, CKD increased IL-6 while reducing the anti-inflammatory cytokines IL-4 and IL-10 in renal tissue [39]. Exercise generally counteracted these abnormalities by increasing IL-10, IL-4, and PTEN and reducing TNF-α, TGF-β, mTOR, and rpS6 [37,39]. In addition, exercise increased HO-1 and iNOS while reducing macrophage accumulation in renal tissue, suggesting improvement in inflammatory and tissue-protective signaling [32]. However, the findings were not completely uniform. Peng et al. reported lower TNF-α but increased PDGFR, phosphorylated-PDGFR, α-SMA, and CD34 in CKD kidney tissue, whereas exercise increased TNF-α and reduced these remodeling-associated signaling markers [33]. Therefore, the overall pattern supports exercise-mediated modulation of renal inflammatory and molecular signaling, but individual cytokines and signaling pathways may respond differently depending on the CKD model and experimental conditions [32,33,37,39].

In skeletal muscle, CKD produced a broader inflammatory response characterized by increased IL-6, TNF-α, NLRP3, ASC, caspase-1, IL-1β, and IL-18, indicating activation of the NLRP3 inflammasome pathway [35]. Exercise markedly reduced all of these inflammatory and inflammasome-related markers, supporting an anti-inflammatory effect of exercise in CKD-associated skeletal muscle dysfunction [35] (Table 3).

#### 3.4.3. Apoptosis and Cell Death Pathways

Evidence for apoptosis was particularly prominent in renal tissue. CKD increased Fas (CD95), Bax, cleaved caspases, μ-calpain, and DNA fragmentation, while reducing Bcl-2 and intramitochondrial cytochrome c, together indicating substantial activation of apoptotic and cell-death pathways [29]. The increase in TUNEL-positive cells further confirmed enhanced renal cell death in CKD [29]. Exercise substantially reversed these abnormalities, decreasing Fas, Bax, cleaved caspases, μ-calpain, and DNA fragmentation while increasing Bcl-2 and intramitochondrial cytochrome c, accompanied by fewer TUNEL-positive cells [29]. These findings indicate that exercise may protect renal tissue by suppressing both upstream death signaling and downstream execution of apoptosis.

A similar anti-apoptotic effect was observed in skeletal muscle. CKD increased Bax and cleaved caspase-3, whereas exercise reduced both markers, suggesting attenuation of apoptotic signaling in skeletal muscle [35]. Overall, the available tissue-specific evidence indicates that exercise has a cell-protective and anti-apoptotic effect in both kidney and skeletal muscle, although renal apoptosis has been characterized more comprehensively than muscle apoptosis [29,35] (Table 3).

#### 3.4.4. Histopathology and Structural Kidney Changes

The histopathological findings consistently demonstrate structural renal injury in experimental CKD. CKD was associated with cortical damage, glomerular injury, glomerular enlargement, edema, collagen deposition, fibrosis, glomerulosclerosis, capsular adhesions, tubular atrophy, tubular cell loss, inflammation, and thickening of the tubular basement membrane [29,33,35,37,38,39]. These findings indicate that CKD produces both glomerular and tubulointerstitial structural damage, with fibrosis and loss of normal renal architecture representing important pathological consequences. Exercise training generally demonstrated a protective effect against these structural abnormalities. Exercise reduced collagen deposition, glomerular volume, and edema [33], attenuated glomerular injury and tubulointerstitial fibrosis [35], and reduced glomerulosclerosis, interstitial fibrosis, inflammation, tubular injury, and overall renal injury scores [37]. Other studies similarly reported reduced fibrosis and preservation of renal cortical or overall renal architecture following exercise [29,34,39]. Exercise also reduced tubular injury and improved renal histological appearance in CKD animals [38]. Collectively, these findings suggest that exercise contributes to preservation of renal structural integrity and attenuation of fibrosis and tissue remodeling in experimental CKD [29,33,34,35,37,38,39] (Table 3).

#### 3.4.5. Muscular Outcomes

The muscular findings demonstrate that CKD produces substantial skeletal muscle abnormalities, although the response to exercise was not completely consistent across studies. CKD reduced muscle mass, grip strength, maximal load capacity, and gastrocnemius and tibialis anterior muscle weights, accompanied by reductions in muscle hypertrophy and regeneration-related markers including MyHC, MyoD, myogenin, and Pax-7 [35,37]. CKD also increased muscle catabolic markers such as Atrogin-1, MuRF-1 and myostatin and impaired mitochondrial-related markers, including mtDNA, ATP, PGC-1α, TFAM and CoxIV [35]. These findings indicate that CKD-associated muscle dysfunction involves muscle wasting, impaired regeneration, increased proteolysis, and mitochondrial dysfunction. Exercise generally improved these abnormalities. Exercise increased grip strength, muscle cross-sectional area, muscle weights, MyHC, MyoD, myogenin and Pax-7 while reducing Atrogin-1, MuRF-1 and myostatin and restoring mitochondrial-related markers such as mtDNA, ATP, PGC-1α, TFAM and CoxIV [35]. Similarly, exercise increased muscle mass and maximal load capacity in another CKD model [37], while improving muscle strength without substantially changing muscle mass in another study [39]. However, one study reported a different response: exercise did not improve EDL torque, relaxation time, or fiber cross-sectional area and was associated with increased EDL muscle catabolism, ubiquitin protein expression, and Atrogin-1 expression, despite unchanged IGF-1, p70 signaling, MyoD, and myogenin [36]. Therefore, while the majority of evidence suggests that exercise attenuates CKD-associated muscle wasting and functional impairment, the response may depend on the exercise protocol, CKD severity, duration of intervention, and specific muscle examined [35,36,37,39] (Table 3).

### 3.5. Pooled Effects of Exercise on Renal Parameters in Chronic Kidney Disease

**Figure 2:**
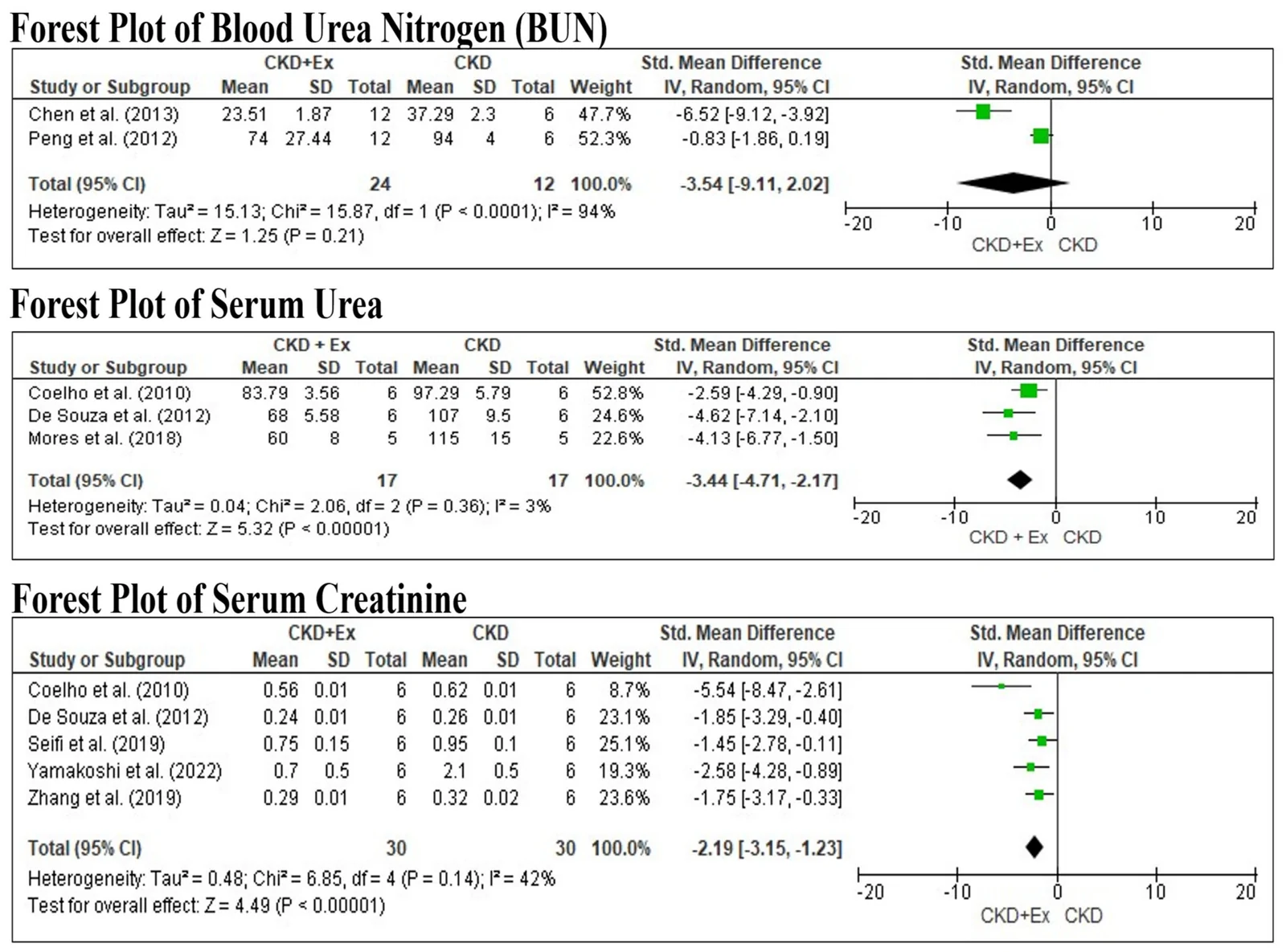
Forest plots depicting the meta-analytic effect of exercise on renal parameters with corresponding confidence intervals, including Blood Urea Nitrogen (BUN), serum urea, and serum creatinine. This figure was generated using Review Manager (RevMan) version 5.4.1. Green squares represent the effect estimates of individual studies, with square size proportional to the study weight; horizontal lines represent 95% confidence intervals (CIs); and black diamonds represent the pooled effect estimates with their 95% CIs. The vertical line at zero represents the line of no effect. Analyses were performed using a random-effects model and are presented as standardized mean differences (SMDs). Chen et al. [29]; Coelho et al. [30]; De Souza et al. [31]; Moraes et al. [32]; Peng et al. [33]; Yamakoshi et al. [34]; Zhang et al. [35]; Seifi et al. [38].

The main meta-analysis demonstrated that exercise had differential effects on the assessed renal biomarkers in patients with CKD. For BUN, the pooled analysis showed a non-significant reduction in the exercise group compared with the CKD control group (SMD = −3.54, 95% CI: −9.11 to 2.02, *p* = 0.21), with very high heterogeneity (I^2^ = 94%), indicating substantial variability among the included studies [29,32]. Similarly, meta-analysis of serum urea incorporating three studies [30,31,32] showed a significant reduction in the exercise group (SMD = −3.44; 95% CI: −4.71 to −2.17; *p* < 0.00001), with minimal heterogeneity (I^2^ = 3%), indicating a consistent effect of exercise on serum urea reduction. Moreover, pooled analysis of serum creatinine based on five studies [30,31,34,35,38] demonstrated a significant reduction favoring the exercise intervention group (SMD = −2.19; 95% CI: −3.15 to −1.23; *p* < 0.00001). Although moderate heterogeneity was observed (I^2^ = 42%), the direction of effect consistently favored exercise across all included studies. The observed heterogeneity may reflect methodological and biological differences among studies, including variations in exercise protocols, intervention duration, and CKD induction models, rather than inconsistency in the therapeutic effects of exercise (Figure 2).

**Figure 3:**
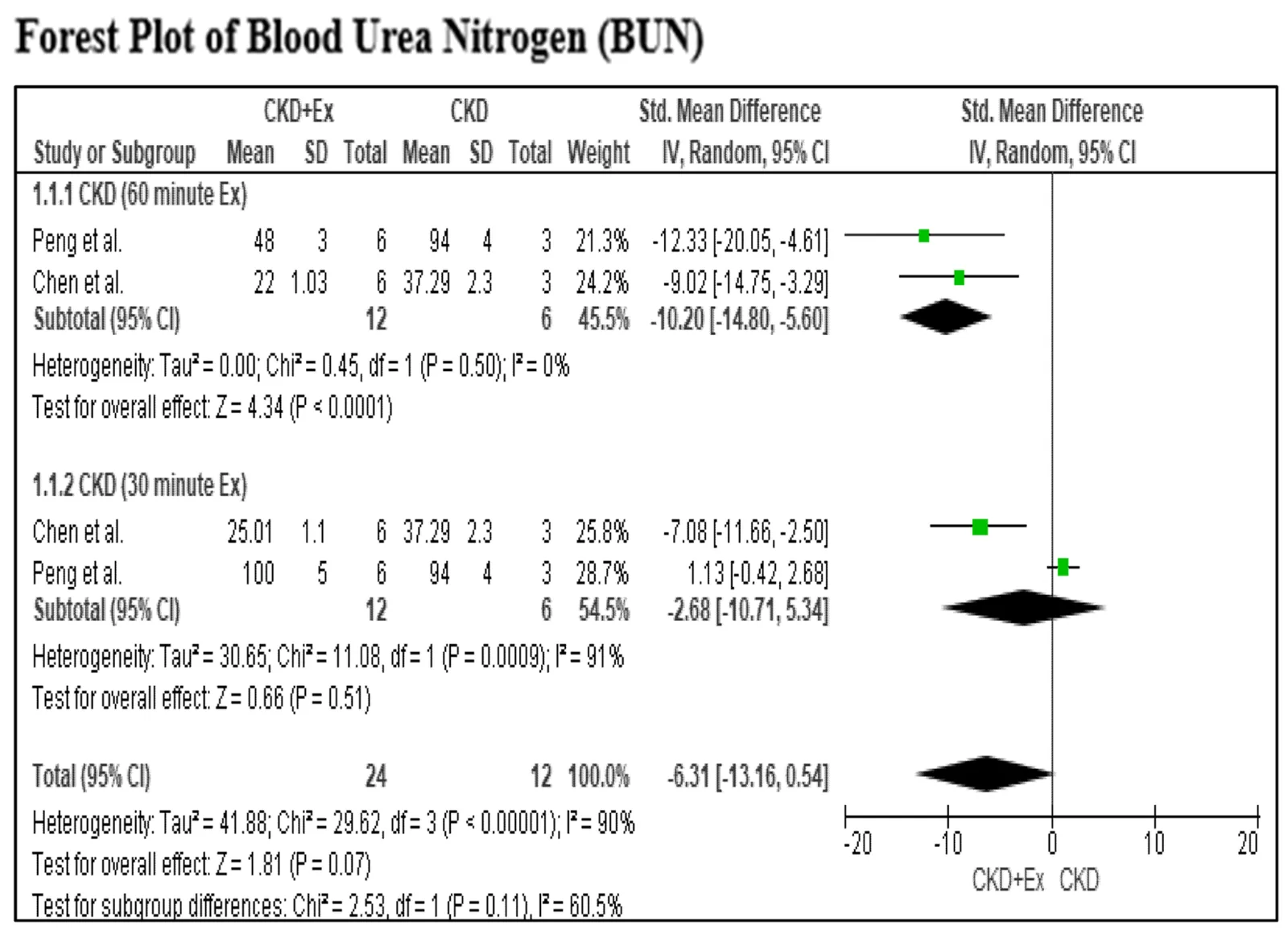
Subgroup analysis of blood urea nitrogen (BUN) according to exercise duration in patients with chronic kidney disease. The analysis compares the effects of 30 min and 60 min exercise sessions between the CKD + exercise and CKD control groups using standardized mean differences (SMDs) with 95% confidence intervals (CIs). This figure was generated using Review Manager (RevMan) version 5.4.1. Green squares represent the effect estimates of individual studies, with square size proportional to the study weight; horizontal lines represent 95% confidence intervals (CIs); and black diamonds represent the pooled effect estimates with their 95% CIs. The vertical line at zero represents the line of no effect. Analyses were performed using a random-effects model and are presented as standardized mean differences (SMDs). Chen et al. [29] and Peng et al. [33].

The subgroup analysis of BUN according to exercise duration showed that 60 min exercise sessions were associated with a significant reduction in BUN (SMD = −10.20, 95% CI: −14.80 to −5.60, *p* < 0.0001), with no observed heterogeneity (I^2^ = 0%). In contrast, the 30 min exercise subgroup did not demonstrate a statistically significant reduction in BUN (SMD = −2.68, 95% CI: −10.71 to 5.34, *p* = 0.51) and showed substantial heterogeneity (I^2^ = 91%). When both subgroups were pooled, the overall effect remained non-significant (SMD = −6.31, 95% CI: −13.16 to 0.54, *p* = 0.07; I^2^ = 90%). Importantly, the test for subgroup differences was not statistically significant (χ^2^ = 2.53, df = 1, *p* = 0.11), indicating that although the 60 min subgroup showed a significant reduction in BUN, the available evidence does not demonstrate a statistically significant difference between 30- and 60 min exercise durations (Figure 3).

### 3.6. Pooled Effects of Exercise on Oxidative Stress Markers

**Figure 4:**
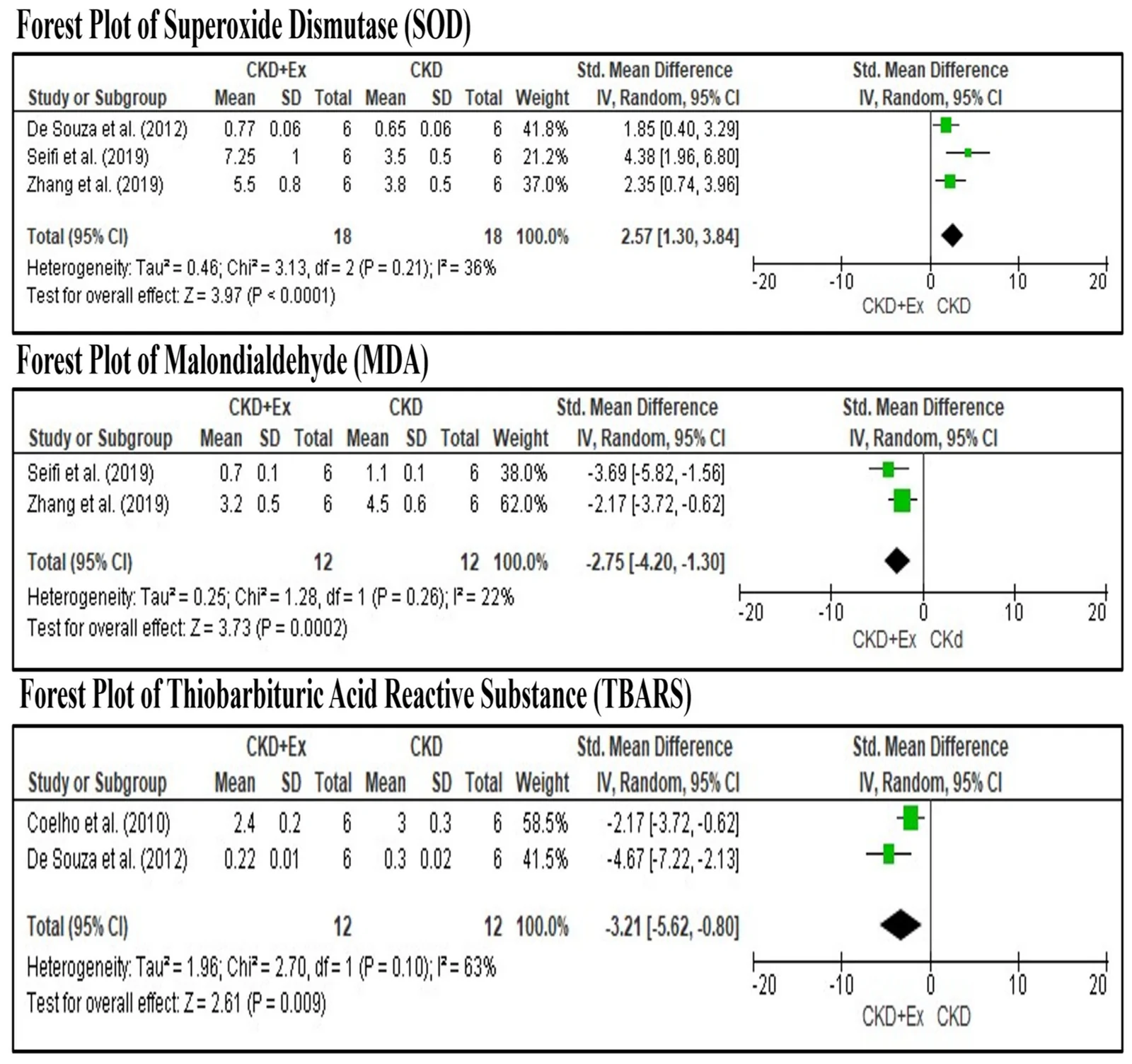
Forest plots depicting the meta-analytic effect of exercise on oxidative stress markers with corresponding confidence intervals, including Superoxide Dismutase (SOD), Malondialdehyde (MDA), and Thiobarbituric Acid Reactive Substances (TBARS). This figure was generated using Review Manager (RevMan) version 5.4.1. Green squares represent the effect estimates of individual studies, with square size proportional to the study weight; horizontal lines represent 95% confidence intervals (CIs); and black diamonds represent the pooled effect estimates with their 95% CIs. The vertical line at zero represents the line of no effect. Analyses were performed using a random-effects model and are presented as standardized mean differences (SMDs). Coelho et al. [30]; De Souza et al. [31]; Zhang et al. [35]; Seifi et al. [38].

Across rat models of CKD, exercise interventions (CKD+EX) demonstrated consistent antioxidant and protective effects, although responses varied according to the assessed biomarker. Meta-analysis of three studies [30,35,38], evaluating SOD levels, demonstrated a statistically significant increase in SOD in the intervention group compared with the sedentary CKD group. Moderate heterogeneity was observed (I^2^ = 36%), indicating acceptable consistency among studies. All included studies favored exercise, suggesting enhanced antioxidant defense capacity in CKD. Similarly, pooled analysis of two studies [35,38] assessing MDA demonstrated a significant reduction in the intervention group with low heterogeneity (SMD = −2.75; 95% CI: −4.20 to −1.30; *p* = 0.0002; I^2^ = 22%). Meta-analysis of two studies [30,31], assessing TBARS, also showed a significant reduction favoring the exercise group (SMD = −3.21; 95% CI: −5.62 to −0.80; *p* = 0.009). However, substantial heterogeneity was observed (I^2^ = 63%), indicating variability across the included studies (Figure 4).

**Figure 5:**
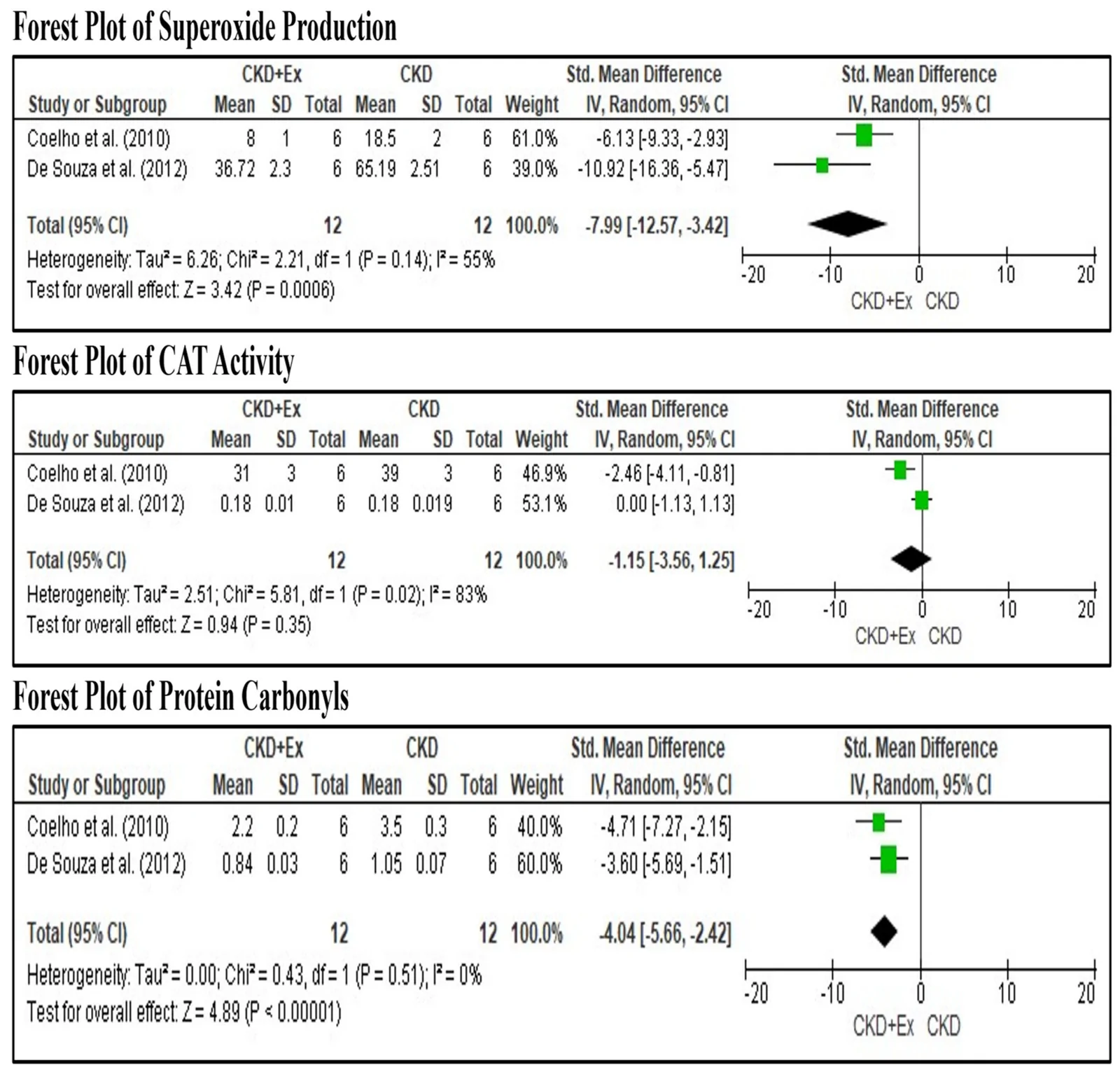
Forest plots depicting the meta-analytic effect of exercise on oxidative stress markers with corresponding confidence intervals, including Superoxide Production, CAT activity, and Protein Carbonyls. This figure was generated using Review Manager (RevMan) version 5.4.1. Green squares represent the effect estimates of individual studies, with square size proportional to the study weight; horizontal lines represent 95% confidence intervals (CIs); and black diamonds represent the pooled effect estimates with their 95% CIs. The vertical line at zero represents the line of no effect. Analyses were performed using a random-effects model and are presented as standardized mean differences (SMDs). Coelho et al. [30]; De Souza et al. [31].

In analyses of superoxide production, the intervention (CKD+EX) group demonstrated a significant reduction in ROS generation (SMD = −7.99; 95% CI: −12.57 to −3.42; *p* = 0.0006), with moderate heterogeneity (I^2^ = 55%) [30,31]. In contrast, catalase (CAT) activity showed a non-significant overall effect (SMD = −1.15; 95% CI: −3.56 to 1.25; *p* = 0.35), accompanied by substantial heterogeneity (I^2^ = 83%) [30,31], suggesting inconsistent antioxidant enzyme responses across studies. Meanwhile, protein carbonyl levels, a marker of oxidative protein damage, were significantly reduced following exercise interventions in CKD rats (SMD = −4.04; 95% CI: −5.66 to −2.42; *p* < 0.00001), with no observed heterogeneity (I^2^ = 0%), indicating a robust and consistent protective effect [30,31]. Collectively, these findings suggest that exercise effectively attenuates oxidative stress in CKD by reducing oxidative damage and ROS generation, although its effects on endogenous antioxidant enzymes such as CAT may vary across experimental models (Figure 5).

## 4. Discussion

**Figure 6:**
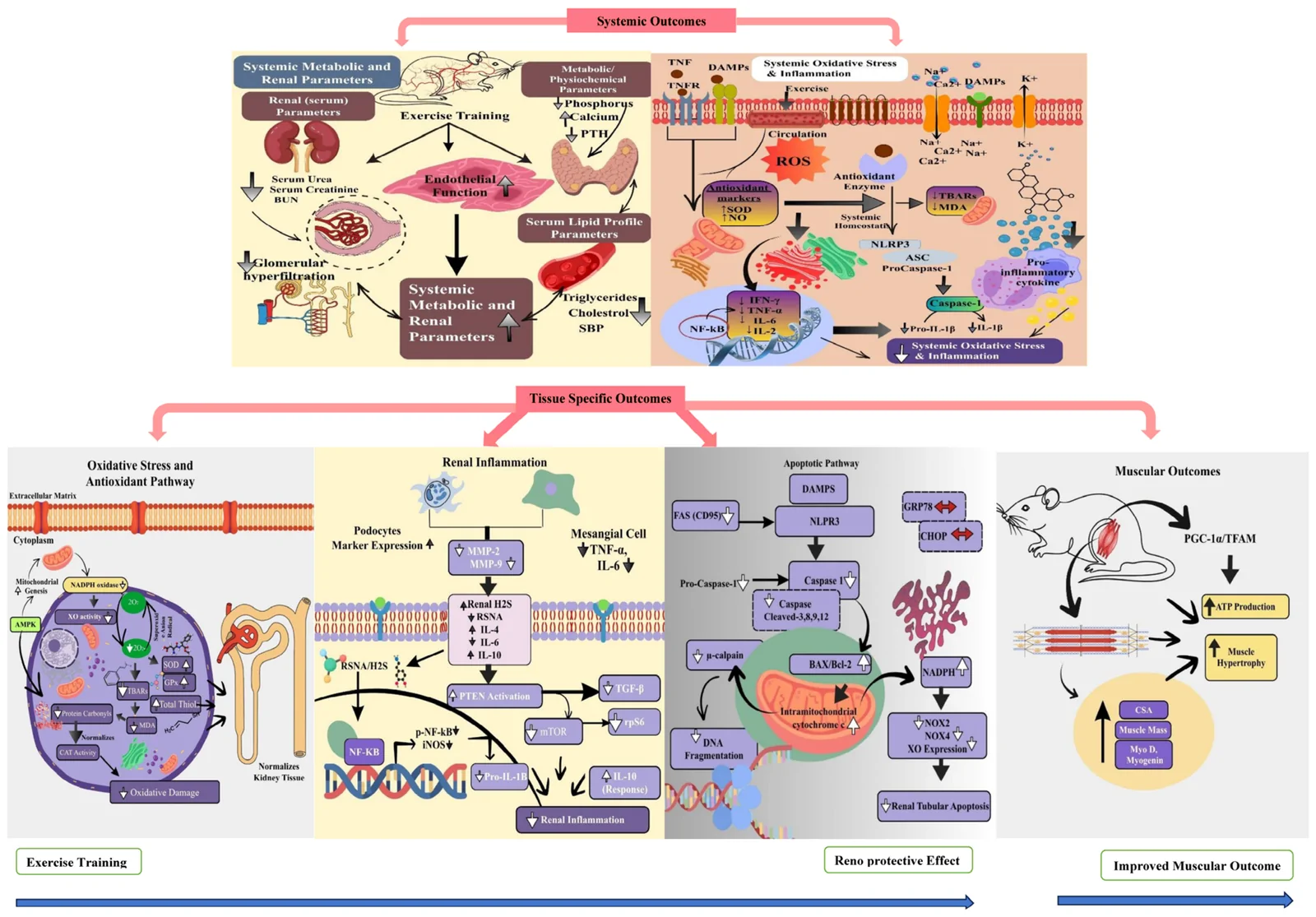
Mechanistic Pathways Underlying the Effect of Exercise Training in an Animal Model of Chronic Kidney Disease. **AMPK** = AMP-activated Protein Kinase; **ASC** = Apoptosis-associated Speck-like Protein containing a CARD; **ATP** = Adenosine Triphosphate; **BAX** = Bcl-2-associated X Protein; **Bcl-2** = B-cell Lymphoma 2; **BUN** = Blood Urea Nitrogen; **CAT** = Catalase; **Ca^2+^** = Calcium ion; **CHOP** = C/EBP Homologous Protein; **DAMPs** = damage-associated molecular patterns; **DNA** = Deoxyribonucleic Acid; **Fas (CD95)** = Fas cell surface death receptor; **GPx** = Glutathione Peroxidase; **GRP78** = Glucose-Regulated Protein 78; **H_2_S** = Hydrogen Sulfide; **HO** = Heme Oxygenase; **IFN-γ** = Interferon-Gamma; **IL-1β** = Interleukin-1 beta; **IL-2** = Interleukin-2; **IL-4** = Interleukin-4; **IL-6** = Interleukin-6; **IL-10** = Interleukin-10; **iNOS** = Inducible Nitric Oxide Synthase; **K^+^** = Potassium ion; **MDA** = Malondialdehyde; **MMP-2** = Matrix Metalloproteinase-2; **MMP-9** = Matrix Metalloproteinase-9; **mTOR** = Mammalian Target of Rapamycin; **MyoD** = Myogenic Differentiation 1; **Na^+^** = Sodium ion; **NADPH** = Nicotinamide Adenine Dinucleotide Phosphate; **NF-κB** = Nuclear Factor kappa B; **NLRP3** = NOD-like Receptor family Pyrin domain-containing 3; **NO** = Nitric Oxide; **NOX2** = NADPH Oxidase 2; **NOX4** = NADPH Oxidase 4; **PGC-1α** = Peroxisome Proliferator-activated Receptor Gamma Coactivator-1 alpha; **PTEN** = Phosphatase and Tensin Homolog; **PTH** = Parathyroid Hormone; **ROS** = Reactive Oxygen Species; **rpS6** = Ribosomal Protein S6; **RSNA** = Renal Sympathetic Nerve Activity; **SBP** = Systolic Blood Pressure; **SOD** = Superoxide Dismutase; **TBARS** = Thio Barbituric Acid Reactive Substances; **TFAM** = Mitochondrial Transcription Factor A; **TGF-β** = Transforming Growth Factor-beta; **TNF-α** = Tumor Necrosis Factor-alpha; **TNFR** = Tumor Necrosis Factor Receptor; **XO** = Xanthine Oxidase. Box-to-box arrows indicate the proposed directional relationships between the depicted pathways and outcomes; ↑ and ↓ shown alongside biomarkers indicate increased and decreased levels or activity, respectively, while ↔ indicates no apparent change. The long blue arrows at the bottom indicate the overall progression from exercise training toward renoprotective effects and improved muscular outcomes. This figure was created using Canva Pro (Canva Pty Ltd.; https://canva.link/uw0c5romsqcuunj, accessed on 28 May 2026). No third-party copyrighted materials requiring additional permission were used.

The present review demonstrates that experimental CKD in rats induces extensive systemic and tissue-specific dysfunction, including progressive renal impairment, oxidative stress, inflammation, apoptosis, and muscular atrophy. The principal finding is that both aerobic and resistance exercise interventions exert predominantly protective effects across these domains, although the magnitude and consistency of these effects vary according to outcome type and disease severity (Figure 6). The most pronounced benefits were observed in oxidative stress reduction, inflammatory modulation, and preservation of renal histopathology, whereas renal biochemical markers and muscular structural outcomes demonstrated greater variability. Regarding renal biochemical parameters, CKD consistently increased BUN, creatinine, urea, and proteinuria, reflecting impaired renal clearance and glomerular dysfunction. Exercise interventions demonstrated relatively consistent reductions in BUN and proteinuria across studies [29,32,33,36,37,38], suggesting improved renal excretory function and attenuation of glomerular permeability impairment (Figure 6).

Serum creatinine and urea, however, demonstrated heterogeneous responses across studies. Several studies [31,32,35] reported significant improvements following exercise interventions, whereas others showed minimal or no changes [31,34]. This variability may reflect differences in CKD severity and residual nephron mass, as serum creatinine is more closely associated with irreversible structural renal injury. These findings suggest that exercise may preferentially improve functional aspects of renal impairment while exerting limited effects on progressive structural damage. Such findings were aligned with previous investigations from experimental and mechanistic studies, exhibiting that exercise training primarily improved renal functional capacity through increased renal blood flow, reduced oxidative stress, and attenuation of inflammatory signaling, rather than reversing established structural injury. In animal models of CKD, aerobic and endurance exercise has been demonstrated to enhance glomerular hemodynamics and antioxidant enzyme activity [25]. Similarly, prior studies also showed that exercise training significantly enhanced renal biochemical function in 5/6 nephrectomized rats, without affecting the structural damage [25]. This interpretation is further supported by kidney functional parameters. CKD-induced reductions in GFR and increases in the kidney weight-to-body weight (KW/BW) ratio indicate progressive nephron loss and compensatory renal hypertrophy.

Resistance exercise was associated with improved GFR [32], suggesting enhanced renal filtration capacity, whereas reductions in the kidney weight-to-body weight (KW/BW) ratio reported by two studies [29,32] indicated attenuation of renal hypertrophy. These outcomes aligned with a prior study that revealed that swimming exercise in rats with experimental chronic renal failure showed significant elevation in GFR relative to the unexercised group [40]. Beyond renal outcomes, CKD was also associated with systemic disturbances, including hematological abnormalities, hypertension, dyslipidemia, electrolyte imbalance, and reduced body weight. Exercise interventions demonstrated generally favorable, although variable, effects on these parameters. Improvements in hematological indices, including increased red blood cell counts and reduced platelet levels, were reported by one study [29], whereas reductions in systolic blood pressure and improvements in lipid profiles were observed in other studies [32,33].

Nevertheless, persistent hypertension in certain CKD models [34] and relatively minor changes in glucose levels [32] suggest that systemic responses to exercise are not entirely uniform and may vary according to disease severity and intervention characteristics. Modulation of oxidative stress emerged as one of the most consistent findings across the included studies. CKD was associated with reduced antioxidant defenses, including SOD and total thiols, alongside increased MDA, TBARS, and ROS–generating enzymes [30,31,34,38]. These alterations were attenuated by exercise interventions, which improved oxidative stress markers and enhanced antioxidant capacity [29,32,34]. Previous studies reported that Regular exercise decreased oxidative stress and increased antioxidant enzymes, such as SOD, catalase, and GPx, in renal tissues [41]. In CKD, exercise might improve immune function and have anti-inflammatory effects [42]. Similarly, a study including Wistar rats showed that regular and progressive aerobic exercise reduces the severity of tubular injury and the intensity of caspase 3 within 48 h after reperfusion [43].

At the tissue level, mitochondrial oxidative balance also improved following exercise interventions, as demonstrated by increased SOD2 expression and reduced MDA levels in muscular tissue [35]. This consistency across experimental models suggests that oxidative stress represents a central and highly responsive therapeutic target of exercise in CKD. Closely associated with oxidative stress, apoptotic pathways were markedly activated in CKD models, characterized by increased expression of Bax, caspases, and μ-calpain, alongside reduced anti-apoptotic proteins such as Bcl-2 [29,34,35]. Exercise interventions attenuated these apoptotic responses, suggesting preservation of cellular integrity and tissue survival. Histopathological findings further supported the structural protective effects of exercise. CKD-induced renal injury, including fibrosis, glomerulosclerosis, tubular injury, and inflammatory infiltration, was consistently ameliorated following exercise interventions [29,32,33,37,38,39]. Such findings aligned with prior studies, which found that regular aerobic exercise and preceding adaptation decrease the structural damage of the kidney, including interstitial edema, mononuclear cell infiltration, and injury of tubular and brush-margin cells [43].

These improvements were observed even in studies reporting only modest changes in biochemical markers, suggesting that structural recovery may precede detectable systemic improvements. This finding highlights the importance of histopathological assessment in evaluating therapeutic efficacy. Exercise also demonstrated consistent modulatory effects on inflammatory and molecular signaling pathways. CKD was associated with elevated pro-inflammatory cytokines, including IL-6, TNF-α, and IFN-γ, alongside activation of fibrotic pathways such as TGF-β and mTOR [32,33,35,37,39]. Exercise interventions reduced pro-inflammatory markers while enhancing anti-inflammatory mediators such as IL-10, indicating a shift toward an anti-inflammatory state. Previous literature has shown that the benefits of aerobic exercise on reducing renal inflammation and fibrosis have been well established. Moderate-intensity treadmill training has been shown to reduce renal inflammatory response and improve renal fibrosis and renal injury in rat models. This is accomplished by blocking the activation of inflammatory bodies called the NOD-like receptor family, caspase recruitment domain-containing 4 (NLRC4), and Toll-like receptor 4 (TLR4)/NF-κB pathway. Similarly, another prior study, which recruited 12 weeks of aerobic exercise, showed the suppression of the inflammatory and fibrotic cascade in the renal cortex of rats [44]. This was reflected by a substantial decrease in the content of inflammation-associated proteins such as IL-6 and cyclooxygenase-2 (COX-2), and less expression of fibrosis-associated proteins including transforming growth factor-β (TGF-β), p-Smad2/3, connective tissue growth factor (CTGF), Matrix Metalloproteinase-9 (MMP-9), and Matrix Metalloproteinase-2 (MMP-2) [44]. Moreover, eight weeks of aerobic exercise was found to have significantly reduced oxidative stress, inflammation, and fibrosis by inhibiting the Nox4/ROS/NF-κB/NLRP3 signaling pathway, thus effectively reducing kidney injury in mice [45]. However, the diversity of molecular markers assessed across studies limited comprehensive pathway-level interpretation. Therefore, although these findings provide experimental support for the involvement of oxidative, inflammatory, apoptotic, and fibrotic pathways, the relative contribution of individual signaling pathways remains incompletely established. Some pathways identified in the included studies should consequently be considered mechanistically supported associations rather than definitive causal pathways, particularly where pathway-specific interventions or direct mechanistic validation were not performed.

Muscular findings further demonstrated that CKD induces muscle wasting, characterized by reduced muscle mass, strength, and mitochondrial activity, alongside increased catabolic signaling. Exercise interventions consistently improved muscle strength, mitochondrial function, and anabolic signaling [32,35,37]. However, gains in muscle mass were less consistent, and several studies reported minimal morphological changes despite functional improvements [36,39]. These findings suggest that functional and metabolic adaptations may precede structural muscular recovery. Such findings were supported by a previous study that resistance training has been demonstrated to be an effective strategy for improving muscle strength in rats with CKD [46].

The methodological quality of the included studies, assessed using the CAMARADES 10-item checklist, ranged from 5 to 7 out of 10, indicating moderate methodological quality across the available evidence. Although the included studies fulfilled several key methodological criteria, important gaps were identified, particularly regarding allocation concealment, blinded outcome assessment, and formal sample size calculation. Allocation concealment was not reported across the included studies, while blinded outcome assessment and reporting of anesthetic use were inconsistently described. These methodological considerations are important when interpreting the observed beneficial effects of exercise, as incomplete methodological safeguards may introduce potential sources of bias and reduce confidence in the magnitude of the estimated effects. Thus, the consistent direction of benefit across several renal, oxidative, inflammatory, histopathological, and muscular outcomes provides supportive evidence for the protective effects of exercise in experimental CKD; however, the findings should be interpreted as promising rather than definitive given the moderate methodological quality of the underlying studies.

### 4.1. Clinical Implication

The findings have potential implications for exercise-based renal rehabilitation and comprehensive CKD management. The observed improvements in oxidative stress, inflammatory signaling, renal histopathology, and skeletal-muscle function suggest that exercise may provide benefits beyond physical conditioning. Accordingly, appropriately prescribed aerobic and resistance exercise may be considered as a component of multidisciplinary CKD care, with exercise intensity and modality individualized according to disease severity, comorbidities, functional capacity, and tolerance. However, because the evidence synthesized in this review was derived exclusively from experimental animal models, these findings should not be interpreted as evidence that exercise prevents or reverses CKD progression in humans. Exercise should therefore complement, rather than replace, established medical management, and well-designed clinical trials are required to determine whether the observed preclinical benefits translate into meaningful improvements in kidney function, physical performance, quality of life, and long-term clinical outcomes.

### 4.2. Limitations and Future Recommendations

Despite the overall beneficial findings, several limitations should be considered. CKD models were highly heterogeneous, including 5/6 nephrectomy and toxin-induced models, which differ in pathophysiology and disease progression. Exercise interventions also varied considerably in type, intensity, and duration, limiting direct comparability and identification of optimal exercise regimens. Furthermore, substantial methodological heterogeneity was observed in the assessment of renal function, oxidative stress, inflammatory biomarkers, and histopathological outcomes, contributing to variability in the reported effects. Several studies lacked detailed reporting of key functional, molecular, and methodological characteristics, including randomization, allocation concealment, and blinding procedures, increasing the risk of bias and reducing reproducibility. Most studies included relatively small sample sizes, and the evidence was derived almost exclusively from rodent models, with a predominance of male animals, thereby limiting the generalizability of the findings to female animals, other species, and ultimately to human populations. In addition, this review has several methodological limitations. Although a comprehensive literature search and rigorous study selection process were undertaken, only published studies written in English were included, which may have introduced language and publication bias. The limited number of studies available for several outcomes restricted the ability to perform subgroup analyses and formally assess publication bias. Moreover, inconsistencies in outcome reporting and incomplete quantitative data prevented the inclusion of some outcomes in the meta-analysis, necessitating qualitative synthesis for certain findings. These limitations should be considered when interpreting the results and highlight the need for more standardized, high-quality preclinical studies to strengthen the evidence base and facilitate future translation into clinical research. Future research should prioritize the translation of preclinical findings into well-designed clinical trials to determine the clinical applicability of exercise interventions in CKD. Large-scale randomized controlled trials involving diverse CKD populations are needed to establish the safety, efficacy, and optimal prescription of exercise across different disease stages. Standardization of exercise protocols, including exercise type, intensity, frequency, and duration, is also necessary to improve comparability and reproducibility across studies. Furthermore, future investigations should incorporate comprehensive outcome measures, including renal functional parameters, molecular biomarkers, quality of life, and physical performance outcomes. Longitudinal studies are particularly important to evaluate the long-term effects of exercise and identify the most effective timing and dosage of interventions during CKD progression. Finally, multidisciplinary approaches integrating physiological, biochemical, and histopathological analyses may further improve mechanistic understanding and support the development of evidence-based clinical guidelines.

## 5. Conclusions

Exercise exerts multi-level protective effects in experimental CKD, with the most consistent benefits observed in oxidative stress reduction, inflammation attenuation, and preservation of renal structure. However, variability in renal biochemical and muscular structural outcomes suggests that exercise primarily slows disease progression rather than reversing advanced renal damage. Collectively, these findings support the potential role of exercise as a promising adjunct therapeutic strategy in CKD management.

## 6. Patient-Friendly Summary

CKD can affect not only the kidneys but also muscles and the body’s ability to control inflammation and oxidative stress. In experimental animal studies, regular exercise generally improved several of these problems, including markers of kidney injury, inflammation, oxidative stress, and muscle function. These findings suggest that exercise may have protective effects in CKD; however, because the evidence comes from animal studies, it is not yet possible to conclude that exercise will prevent kidney disease progression in people. Exercise should therefore be appropriately individualized and used alongside, rather than instead of, standard medical care.

## Figures and Tables

**Table 1:** The CAMARADES Checklist.

References	1	2	3	4	5	6	7	8	9	10	Total
Chen et al. [29]	✓	✓	✓			✓	✓		✓	✓	7/10
Coelho et al. [30]	✓	✓	✓				✓		✓	✓	6/10
De Souza et al. [31]	✓	✓	✓			✓	✓		✓	✓	7/10
Moraes et al. [32]	✓	✓	✓		✓		✓		✓	✓	7/10
Peng et al. [33]	✓	✓	✓				✓		✓	✓	6/10
Yamakoshi et al. [34]	✓	✓	✓			✓	✓		✓	✓	7/10
Zhang et al. [35]	✓		✓		✓		✓		✓	✓	6/10
Organ et al. [36]	✓		✓				✓		✓	✓	5/10
Saud et al. [37]	✓	✓	✓		✓		✓		✓	✓	7/10
Seifi et al. [38]	✓	✓	✓			✓	✓		✓	✓	7/10
Souza et al. [39]	✓	✓	✓			✓	✓		✓	✓	7/10

**1.** Publication in a peer-reviewed journal. **2.** Statement of control of temperature. **3.** Randomization of treatment or control. **4.** Allocation concealment. **5.** Blinded assessment of outcome. **6.** Avoidance of anesthetics with marked intrinsic properties. **7.** Use of animals with chronic kidney disease. **8.** Sample size calculation. **9.** Statement of compliance with regulatory requirements. **10.** Statement regarding possible conflicts of interest. **✓**—yes.

**Table 2:** Study Characteristics and Exercise-Induced Systemic Effects in Experimental Chronic Kidney Disease Models.

Study Characteristics					Outcomes Based on the Systemic Level					
References	Sample	Exercise Type	Exercise Parameters	Sample Size	Renal Parameters (Serum/Plasma/Urine)	Kidney Functional (Systemic)	Physiological and Physicochemical (Systemic)	Oxidative Stress & Antioxidant Markers (Systemic)	Inflammatory & Molecular Signaling Markers (Systemic)	Histopathology & Structural Kidney Changes (Systemic)
Chen et al. [29]	**S:** Sprague-Dawley rats **Sex:** Male **A**: 4 weeks old **W:** 225–250 g **Model:** DRCKD	Treadmill training	**D:** 30–60 min/session (pooled exercise group) F: 3 days/week **P:** 11 weeks	**CKD:** N = 6 rats **CKD+EX:** N = 6 rats	**CKD:** ↑ BUN **CKD+EX:** ↓ BUN	**CKD:** ↑ KW/BW **CKD+EX:** ↓ KW/BW	**CKD:** ↓ RBC ↑ platelets ↑ WBC ↑ cholesterol ↑ triglycerides **CKD+EX:** ↑ RBC ↓platelets, WBC partially normalized, ↑ cholesterol ↑ triglycerides	**CKD:** ↓ SOD, ↑ MDA **CKD+EX:** ↑ SOD, ↓ MDA	Not assessed	Not assessed
Coelho et al. [30]	**S:** Wistar rats, **Sex:** Male **A:** 3 months old **W**: 250–300 g **Model:** 5/6 nephrectomy	Treadmill running	**Speed:** up to 1 km/h **D:** 50 min/day **F:** 5 days/week **P:** 8 weeks	**CKD:** N = 6 rats **CKD+EX:** N = 6 rats	**CKD:** ↑ Urea, ↑ Creatinine **CKD+EX:** ↔ Urea, ↔Creatinine	Not assessed	Not assessed	Not assessed	Not assessed	Not assessed
De Souza et al. [31]	**S**: Wistar rats **Sex**: Male **A:** 3 months old **W:** 250–300 g **Model**: 5/6 nephrectomy	Treadmill running	**Speed:** 13–17 m/min **D:** 50 min/day **F:** 5 days/week **P:** 8 weeks	**CKD:** N = 6 rats **CKD+EX:** N = 6 rats	**CKD:** ↑ Urea, ↑ Creatinine, **CKD+EX:** ↓ Urea, ↔ Creatinine,	Not assessed	Not assessed	Not assessed	Not assessed	Not assessed
Moraes et al. [32]	**S**: Wistar rats **Sex:** Male **A**: 8 weeks old **W:** 230–250 g **Model:** 5/6 nephrectomy	Resistance Exercise: Vertical ladder climbing	**D:** 8 weeks, progressive load based on CLmax **F:** 3 days/week, non-consecutive days.	**CKD:** N = 5 rats **CKD+EX:** N = 5 rats	**CKD:** ↑ Urea, ↑ Creatinine, ↑ Uprot, **CKD+EX:** ↓ Urea, ↓ Creatinine, ↓ Uprot,	**CKD:** ↓ GFR **CKD+EX:** ↑ GFR,	**CKD:** ↑ SBP, ↑ Lipids, ↑ Lactate, ↔ Glucose, ↑ Na^+^/K^+^ ↓ BW **CKD+EX:** ↓ SBP, ↓ Lipids, ↓ Lactate, ↔ Glucose, ↓ Na^+^/K^+^, ↑ BW	**CKD:** ↓ NO, ↔ SOD **CKD+EX:** ↑ NO, ↑ SOD	**CKD:** ↑ IFN-γ, ↑ TNF-α, ↑ IL-2, ↑ IL-6 **CKD+EX:** ↓ IFN-γ, ↓ TNF-α, ↓ IL-2, ↓ IL-6	Not assessed
Peng et al. [33]	**S:** Sprague-Dawley rats **Sex:** Male **A:** 4 weeks old **W**: 220–250 g **Model:** DRCKD	Swimming Exercise	**D**: 30 or 60 min **F:** 3 days/week **P =** 11 weeks	**CKD:** N = 6 rats **CKD+EX:** N = 6 rats	**CKD:** ↑ BUN, ↑ Creatinine, ↑ Proteinuria **CKD+EX:** ↓ BUN, ↔ Creatinine, ↓ proteinuria	**CKD:** ↑ KW/BW **CKD+EX:** ↓ KW/BW	**CKD:** ↓ BW **CKD+EX:** ↑ BW	**CKD:** ↓ SOD, ↑ TBARS **CKD+EX:** ↑ SOD, ↓TBARS	**CKD:** ↑ IL-6, ↑ MMP-2, ↑ MMP-9 **CKD+EX:** ↓ IL-6, ↓ MMP-2, ↓ MMP-9	Not assessed
Yamakoshi et al. [34]	**S**: Sprague–Dawley rats, **Sex:** Male **A**: 6 weeks old **Model**: 5/6 nephrectomy	Treadmill running	**Speed:** 20 m/min **D:** 60 min/day **F:** 5 days/week **12** weeks	**CKD:** N = 6 rats **CKD+EX:** N = 6 rats	**CKD:** ↑ creatinine ↑ Uprot **CKD+EX:** ↓ creatinine, ↓ Uprot	Not assessed	**CKD:** ↓ BW, ↑ SBP **CKD+EX:** ↔ BW, ↑ SBP	**CKD:** ↑ MDA **CKD+EX:** ↓ MDA #	Not assessed	Not assessed
Zhang et al. [35]	**S:** C57BL/6J mice **Sex:** Male **A:** 8–12 weeks old **Model:** 5/6 nephrectomy	Aerobic exercise (wheel running)	**Speed**: 6.5 m/min **D**: 1 h/day **F**: 5 days/week **P**: 8 weeks	**CKD:** N = 6 mice **CKD+EX:** N = 6 mice	**CKD:** ↑ Creatinine, ↑ BUN, **CKD+EX:** ↓ Creatinine #, ↓ BUN #	Not assessed	**CKD:** ↓ BW **CKD+EX:** ↑ BW #.	Not assessed	Not assessed	Not assessed
Organ et al. [36]	**S:** Cy/+ rats **Sex:** Not specified **A:** 25 weeks **Model:** Progressive CKD (Cy/+ rat)	Treadmill running	**Speed:** 8 m/min → 18 m/min **D**: 60 min/session **F:** 5 days/week **P:** 10 weeks	**CKD:** N = 8 rats **CKD+EX:** **N** = 8 rats	**CKD:** ↑ BUN **CKD+EX:** ↔ BUN	Not assessed	**CKD**: ↑ Phosphorus; ↑ PTH; ↓ Ca **CKD+EX:** ↓ Phosphorus; ↔ PTH; ↑Ca	Not assessed	Not assessed	Not assessed
Saud et al. [37]	**S:** Wistar rats; **Sex:** Male; **W:** 230–250 g; **Model:** 5/6 nephrectomy CKD	Resistance Exercise Training	**D**: 8–12 climbs/session with progressive load **F:** 5 days/week **P:** 8 weeks	**CKD:** N = 6 rats **CKD+EX:** N = 7 rats	**CKD**: ↑ BUN, ↑ Uprot, ↑ creatinine **CKD+EX:** ↓ BUN, ↓ Uprot, ↓ creatinine	Not assessed	**CKD:** ↑ SBP ↓ BW **CKD+EX:** ↑ BW	Not assessed	Not assessed	Not assessed
Seifi et al. [38]	**S:** Wistar rats **Sex**: Male **W:** 250–300 g **Model**: 5/6 nephrectomy CKD	Treadmill exercise	**Speed**: 18 m/min **P:** 8 weeks	**CKD:** N = 6 rats **CKD+EX:** N = 6 rats	**CKD:** ↑ creatinine; ↑ BUN **CKD+EX:** ↓ creatinine; ↓ BUN	Not assessed	**CKD:** ↑ SBP **CKD+EX:** ↓ SBP	Not assessed	Not assessed	Not assessed
Souza et al. [39]	**S:** Munich-Wistar rats, **Sex**: Male, **A**: 8 weeks old, **W:** 240 ± 20 g, **Model:** 5/6 nephrectomy CKD	Resistance training	**D:** 12 min/session 3 days/week **F**: 8–12 dynamic ladder climbs per session **P**: 10 weeks	**CKD:** N = 5 rats **CKD+EX:** N = 5 rats	**CKD:** ↓ CK levels, **CKD+EX:** ↑ CK levels	Not assessed	**CKD:** ↔ BW **CKD+EX:** ↔ BW	Not assessed	Not assessed	Not assessed

**Control group** = CKD (Sedentary); **Intervention group** = CKD+EX. **S**, species; **A**, age; **W**, weight; **D**, duration per session; **F**, frequency; **P**, program duration; **DRCKD**, doxorubicin-induced chronic kidney disease; **CKD**, chronic kidney disease; **CKD+EX**, chronic kidney disease with exercise; **CLmax**, maximum carrying load; **BUN**, blood urea nitrogen; **Uprot**, urinary protein; **GFR**, glomerular filtration rate; **KW/BW**, kidney weight-to-body weight ratio; **RBC**, red blood cells; **WBC**, white blood cells; **SBP**, systolic blood pressure; **BW**, body weight; **Na^+^/K^+^**, sodium/potassium; **SOD**, superoxide dismutase; **MDA**, malondialdehyde; **MMP-2**, Matrix Metalloproteinase-2; **MMP-9**, Matrix Metalloproteinase-9; **TBARS**, thiobarbituric acid reactive substances; **NO**, nitric oxide; **IFN-γ**, interferon gamma; **TNF-α**, tumor necrosis factor-alpha; **IL**, interleukin; **PTH**, parathyroid hormone; **Ca**, calcium; **CK**, creatine kinase. ↑, ↓, and ↔ indicate an increase, decrease, and no significant change, respectively, relative to the corresponding comparator group, as reported by the original study; these symbols represent directionality only and do not inherently indicate improvement or deterioration; **#**, partial reversal.

**Table 3:** Exercise-Induced Tissue-Specific Outcomes in Experimental Chronic Kidney Disease Models.

Tissue-Specific Outcomes						
References	Sample Size	Oxidative Stress & Antioxidant Markers (Tissue)	Inflammatory & Molecular Signaling Markers (Tissue)	Apoptosis & Cell Death Pathways (Tissue)	Histopathology & Structural Kidney Changes (Tissue)	Muscular Outcomes (Tissue)
Chen et al. [29]	**CKD:** N = 6 rats **CKD+EX:** N = 6 rats	Not assessed	**CKD *(Kidney tissue):*** ↑ Fas (CD95); ↔ GRP78; ↔ CHOP **CKD+EX *(Kidney tissue):*** ↓ Fas (CD95) ↔ GRP78; ↔ CHOP	**CKD *(Kidney tissue):*** ↑ Bax; ↓ Bcl-2; ↓ intramitochondrial cytochrome-c; ↑ caspase-9 cleaved, 3,8,12; ↑ μ-calpain; ↑ DNA fragmentation **CKD+EX *(Kidney tissue):*** ↓ Bax; ↑ Bcl-2; ↑intramitochondrial cytochrome c; ↓ caspase-9, cleaved, 3,8,12; ↓ μ-calpain; ↓ DNA fragmentation	**CKD *(Kidney tissue):*** ↑ TUNEL-positive cells, Marked renal cortical damage **CKD+EX *(Kidney tissue):*** ↓ TUNEL-positive cells; preservation of renal cortical architecture	Not assessed
Coelho et al. [30]	**CKD:** N = 6 rats **CKD+EX:** N = 6 rats	**CKD *(Kidney tissue):*** ↑ Superoxide, ↑ TBARS, ↑ Protein carbonyls, ↓ Total thiols, ↑ SOD, ↑ CAT **CKD+EX *(Kidney tissue):*** ↓ Superoxide, ↓ TBARS, ↓ Protein carbonyls, ↑ Total thiols, ↓ SOD, ↓ CAT	Not assessed	Not assessed	Not assessed	Not assessed
De Souza et al. [31]	**CKD:** N = 6 rats **CKD+EX:** N = 6 rats	**CKD *(Kidney tissue):*** ↑ Superoxide, ↔ SOD, ↔ CAT, ↔ GPx, ↑TBARS/Protein carbonyl **CKD+EX *(Kidney tissue):*** ↓ Superoxide, ↑ SOD, ↔ CAT, ↑ GPx, ↓ TBARS/Protein carbonyl	Not assessed	Not assessed	Not assessed	Not assessed
Moraes et al. [32]	**CKD:** N = 5 rats **CKD+EX:** N = 5 rats	Not assessed	Not assessed	Not assessed	**CKD *(Kidney tissue):*** ↑ Fibrosis, ↑ Macrophage, ↓ HO-1, ↓ iNOS **CKD+EX *(Kidney tissue****):* ↓ Fibrosis, ↓ Macrophage, ↑ HO-1, ↑ iNOS	**CKD (Muscle tissue):** ↓ Muscle mass (soleus/plantaris/EDL) **CKD+EX (Muscle tissue):** ↑ Muscle mass (soleus/plantaris/EDL)
Peng et al. [33]	**CKD:** N = 6 rats **CKD+EX:** N = 6 rats	Not assessed	**CKD *(Kidney tissue)****:* ↓ TNF-α, ↑ PDGFR, ↑ p-PDGFR, ↑ α-SMA, ↑ CD34 **CKD+EX *(Kidney tissue):*** ↑ TNF-α, ↓ PDGFR, ↓ p-PDGFR, ↓ α-SMA, ↓ CD34	Not assessed	**CKD:** ***Kidney tissue (cortex & cortex–medulla junction):*** ↑ Collagen deposition, ↑ Glomerular volume, ↑ Edema **CKD+EX:** ***Kidney tissue (cortex & cortex–medulla junction):*** ↓ Collagen deposition, ↓ Glomerular volume, ↓ Edema	Not assessed
Yamakoshi et al. [34]	**CKD:** N = 6 rats **CKD+EX:** N = 6 rats	**CKD *(Kidney tissue):*** ↑ Renal NADPH oxidase activity, ↑ XO activity, ↑ Nox2, ↑ Nox4, ↑ XO expression **CKD+EX *(Kidney tissue):*** ↓ Renal NADPH oxidase activity #, ↓ XO activity #, ↓ Nox2 #, ↓ Nox4 #, ↓ XO expression #	Not assessed	Not assessed	**CKD *(Kidney tissue):*** ↑ Cortical damage, renal dysfunction **CKD+EX *(Kidney tissue):*** Preservation of renal cortical structure #	Not assessed
Zhang et al. [35]	**CKD:** N = 6 mice **CKD+EX:** N = 6 mice	**CKD *(Muscle tissue):*** ↓ SOD2, ↑ MDA **CKD+EX *(Muscle tissue):*** ↑ SOD2#, ↓ MDA #	**CKD *(Muscle tissue):*** ↑ IL-6, ↑ TNF-α, ↑ NLRP3, ↑ ASC, ↑ Caspase-1, ↑ IL-1β, ↑ IL-18 **CKD+EX *(Muscle tissue):*** ↓ IL-6 #, ↓ TNF-α #, ↓ NLRP3 #, ↓ ASC #, ↓ caspase-1 #, ↓ IL-1β #, ↓ IL-18 #	**CKD *(Muscle tissue):*** ↑ Bax, ↑ Cleaved caspase-3 **CKD+EX *(Muscle tissue):*** ↓ Bax#, ↓ Cleaved caspase-3 #	**CKD *(Kidney tissue):*** ↑ Glomerular injury, ↑ Tubulointerstitial fibrosis **CKD+EX *(Kidney Tissue):*** ↓ Glomerular injury #, ↓ Tubulointerstitial fibrosis #	**CKD *(Muscle tissue):*** ↓ Grip strength, ↓ CSA gastrocnemius, ↓ MyHC, ↓ MyoD, ↓ Myogenin, ↓ Pax-7, ↑ Atrogin-1, ↑ MuRF-1, ↑ Myostatin, ↓ mtDNA, ↓ ATP, ↓ PGC-1α, ↓ TFAM, ↓ CoxIV, ↓ Gastrocnemius weight, ↓ Tibialis anterior weight **CKD+EX *(Muscle tissue):*** ↑ grip strength #, ↑ CSA gastrocnemius #, ↑ MyHC #, ↑ MyoD #, ↑ Myogenin #, ↑ Pax-7 #, ↓ Atrogin-1 #, ↓ MuRF-1 #, ↓ Myostatin #, ↑ mtDNA #, ↑ ATP #, ↑ PGC-1α #, ↑ TFAM #, ↑ CoxIV #, ↑Gastrocnemius weight, ↑ Tibialis muscle weights #
Organ et al. [36]	**CKD:** N = 8 rats **CKD+EX:** N = 8 rats	Not assessed	Not assessed	Not assessed	Not assessed	**CKD *(Muscle tissue):*** Normal EDL muscle protein turnover marker/torque and cross-sectional area **CKD+EX *(Muscle tissue):*** ↔ Maximum EDL muscle torque ↔ Half-relaxation time ↔ EDL Muscle fiber cross-sectional area ↑ EDL muscle catabolism, ↑ Ubiquitin protein expression (~50%) ↑ Atrogin-1 gene expression, ↔ IGF-1 expression ↔ p70 signaling pathway ↓ Pax-7 expression ↔ MyoD, ↔ Myogenin
Saud et al. [37]	**CKD:** N = 6 rats **CKD+EX:** N = 7 rats	Not assessed	**CKD *(Kidney tissue):*** ↓ IL-10, ↑ TNF-α, ↑ TGF-β, ↑ mTOR, ↑ rpS6, ↓ PTEN. **CKD+EX *(Kidney tissue):*** ↑ IL-10, ↓ TNF-α, ↓ TGF-β, ↓ mTOR, ↓ rpS6, ↑ PTEN	Not assessed	**CKD *(Kidney tissue):*** ↑ glomerulosclerosis, ↑ capsular adhesions, ↑ interstitial fibrosis, ↑ inflammation, ↑ tubular atrophy, ↑ tubular cell loss, ↑ thickened tubular basement membrane. **CKD+EX (*kidney tissue):*** ↓ renal injury score, ↓ glomerulosclerosis, ↓ interstitial fibrosis, ↓ inflammation, ↓ renal architecture	**CKD *(Muscle tissue):*** ↓ muscle mass (EDL/tibialis anterior/plantaris/soleus), ↓ maximal weight carried. **CKD+EX *(Muscle tissue):*** ↑ muscle mass (EDL/tibialis anterior/plantar/soleus), ↑ maximal load capacity
Seifi et al. [38]	**CKD:** N = 6 rats **CKD+EX:** N = 6 rats	**CKD *(Kidney tissue):*** ↑ MDA; ↓ SOD **CKD+EX *(Kidney tissue):*** ↓ MDA; ↑ SOD	**CKD *(Kidney tissue):*** ↓ renal H_2_S; ↑ RSNA **CKD+EX *(Kidney tissue):*** ↑ renal H_2_S; ↓ RSNA	Not assessed	**CKD *(Kidney tissue):*** ↑ tubular injury; ↓ renal histology; preservation of renal structure **CKD+EX *(Kidney tissue):*** ↓ tubular injury; ↑ renal histology; preservation of renal structure	Not assessed
Souza et al. [39]	**CKD:** N = 5 rats **CKD+EX:** N = 5 rats	Not assessed	**CKD *(Kidney tissue):*** ↑ IL-6, ↓ IL-4, ↓ IL-10 **CKD+EX *(Kidney tissue):*** ↓ IL-6, ↑ IL-4, ↑ IL-10	Not assessed	**CKD *(Kidney tissue):*** ↑ renal fibrosis, ↑ collagen accumulation/fibrosis **CKD+EX *(Kidney tissue):*** ↓ renal fibrosis, renal architecture preserved	**CKD *(Muscle tissue):*** ↓ soleus/gastrocnemius/quadriceps muscle strength ↔ muscle mass **CKD+EX *(Muscle tissue):*** ↑ soleus/gastrocnemius/quadriceps muscle strength, ↔ muscle mass

**Control group** = CKD (Sedentary); **Intervention group** = CKD+EX. **CKD**, chronic kidney disease; **CKD+EX**, chronic kidney disease with exercise; **Ex/EX**, exercise; **CSA**, cross-sectional area; **EDL**, extensor digitorum longus muscle; **SOD**, superoxide dismutase; **SOD2**, mitochondrial superoxide dismutase; **CAT**, catalase; **GPx**, glutathione peroxidase; **MDA**, malondialdehyde; **TBARS**, thiobarbituric acid reactive substances; **NADPH oxidase**, nicotinamide adenine dinucleotide phosphate oxidase; **XO**, xanthine oxidase; **NO**, nitric oxide; **HO-1**, heme oxygenase-1; **iNOS**, inducible nitric oxide synthase; **H_2_S**, hydrogen sulfide; **RSNA**, renal sympathetic nerve activity; **Bax**, Bcl-2-associated X protein; **Bcl-2**, B-cell lymphoma 2; **μ-calpain**, micro-calpain; **TUNEL**, terminal deoxynucleotidyl transferase dUTP nick-end labeling; **Fas (CD95)**, Fas cell surface death receptor; **GRP78**, glucose-regulated protein 78; **CHOP**, C/EBP homologous protein; **Nox2/Nox4**, NADPH oxidase isoforms 2 and 4; **IL**, interleukin; **TNF-α**, tumor necrosis factor-alpha; **TGF-β**, transforming growth factor-beta; **mTOR**, mammalian target of rapamycin; **rpS6**, ribosomal protein S6; **PTEN**, phosphatase and tensin homolog; **PDGFR**, platelet-derived growth factor receptor; **p-PDGFR**, phosphorylated platelet-derived growth factor receptor; **α-SMA**, alpha-smooth muscle actin; **CD34**, cluster of differentiation 34; **NLRP3**, NLR family pyrin domain containing 3; **ASC**, apoptosis-associated speck-like protein containing a CARD; **MyHC**, myosin heavy chain; **MyoD**, myogenic differentiation 1; **Pax-7**, paired box 7; **MuRF-1**, muscle RING finger 1; **PGC-1α**, peroxisome proliferator-activated receptor gamma coactivator 1-alpha; **TFAM**, mitochondrial transcription factor A; **CoxIV**, cytochrome c oxidase subunit IV; **IGF-1**, insulin-like growth factor 1; **p70**, p70 ribosomal S6 kinase. ↑, ↓, and ↔ indicate an increase, decrease, and no significant change, respectively, relative to the corresponding comparator group, as reported by the original study; these symbols represent directionality only and do not inherently indicate improvement or deterioration; **#**, partial reversal.

## Data Availability

No new data were created or analyzed in this study. Data sharing is not applicable to this article.

## Supplementary Materials

The following supporting information can be downloaded at: https://www.mdpi.com/article/10.3390/ijms27177830/s1.
